# Effects of *Panax notoginseng* Water Extract on Immune Responses and Digestive Enzymes in White Shrimp *Litopenaeus vannamei*

**DOI:** 10.3390/ani13071131

**Published:** 2023-03-23

**Authors:** Ya-Ting Chen, Chia-Ling Kuo, Chih-Chung Wu, Chun-Hung Liu, Shu-Ling Hsieh

**Affiliations:** 1 Department of Seafood Science, National Kaohsiung University of Science and Technology, Kaohsiung 81157, Taiwan; 2 Department of Food and Nutrition, Providence University, Taichung 43301, Taiwan; 3 Department of Aquaculture, National Pingtung University of Science and Technology, Pingtung 91201, Taiwan

**Keywords:** *Panax notoginseng*, immune responses, digestive enzymes, *Litopenaeus vannamei*

## Abstract

**Simple Summary:**

*Panax notoginseng* (Burk) F. H. Chen is a traditional Chinese herbal medicine commonly used in clinical applications. This study examined the effects of the *Panax notoginseng* water extract (PNWE) on the immune responses and digestive enzyme activity of *Litopenaeus vannamei* (*L. vannamei*). The results showed that different concentrations of PNWE significantly increased the total haemocyte count, granular haemocytes, emi-granular haemocytes, phenoloxidase and respiratory burst activity, phagocytic ratio, and the phagocytic index of *L. vannamei*. In addition, PNWE also significantly increased the chymotrypsin, trypsin, and amylase activity of *L. vannamei*. Additionally, different concentrations of PNWE significantly reduced the *Vibrio* numbers in the intestine without damaging the hepatopancreas and intestine tissues. These results indicated that PNWE improves the immune responses of *L. vannamei* by increasing the haemocyte count and regulating intestinal digestive enzymes.

**Abstract:**

*Panax notoginseng* (Burk) F. H. Chen is a traditional Chinese herbal medicine commonly used in clinical applications. This study examined the effects of the *Panax notoginseng* water extract (PNWE) on the immune responses and digestive enzyme activity of *Litopenaeus vannamei* (*L. vannamei*). The PNWE (50, 100, and 200 μg (g shrimp)^−1^) was injected into *L. vannamei* to analyze the immune response parameters, including the total haemocyte count (THC), granular haemocytes (GC), semi-granular haemocytes (SGC), hialin haemocyte (HC), the respiratory burst (RB), the phagocytic ratio (PR), the phagocytic index (PI), and phenoloxidase (PO). We evaluated the activity of the intestinal digestive enzymes (trypsin, chymotrypsin, amylase, and lipase), the histopathology, and the intestine *Vibrio* numbers. The results showed that different concentrations of the PNWE significantly increased THC, GC, SGC, PO and RB activity, the PR, and the PI of *L. vannamei* while reducing the HC. In addition, the PNWE also significantly increased the chymotrypsin, trypsin, and amylase activity of *L. vannamei.* Furthermore, 50 µg (g shrimp)^−1^ of PNWE regulated the lipase activity. Additionally, different concentrations of the PNWE significantly reduced the *Vibrio* numbers in the intestine without damaging the hepatopancreas and intestine tissues. These results indicate that the PNWE improves the immune responses of *L. vannamei* by increasing the haemocyte count and regulating intestinal digestive enzymes.

## 1. Introduction

Chinese herbal medicines, such as *Scutellaria baicalensis* [1], *Boesenbergia rotunda* [2], and *Andrographis paniculate* [3], can serve as growth promoters, immunomodulators, and antibacterial agents in aquatic animals due to their positive effects on improving growth, immunity, and disease resistance. Chinese herbal medicine not only lowers the cost of preventive medicine but it is more biodegradable than synthetic molecules, poses no threat to the environment, and it is unlikely to give rise to drug resistance in patients [4]. Therefore, using Chinese herbal medicine to modulate the immune system of aquatic animals may be a valuable strategy for managing disease in aquaculture.

*Panax notoginseng* (Burk) F. H. Chen belongs to *Araliaceae* and the genus *Panax* [5,6], primarily produced in Japan, Myanmar, and the southwestern regions of China [7,8]. More than 200 active substances, such as saponins, polyacetylenes, sterols, volatile oils, flavonoids, polysaccharides, cyclopeptides, and amino acids, have been identified from *Panax notoginsens* [5]. Studies on aquatic animals have demonstrated that the *Panax notoginseng* extract enhances the growth of hybrid groupers (*Epinephelus lanceolatus* ♂ × *Epinephelus fuscoguttatus* ♀) as well as their antioxidant capacity [9]. However, no research has investigated the impact of *Panax notoginseng* on the immune responses and digestive enzymes in shrimp intestines.

Haemocytes play a major role in the immune system of shrimp. They primarily control the cellular defense mechanism by releasing defense factors that protect the shrimp’s body from pathogen attacks [10]. In haemocytes, the prophenoloxidase system (proPO system) and phagocytosis are of particular importance [10]. Phenoloxidase (PO) is mainly activated by the proPO system, which produces melanin with bactericidal properties; these properties promote superficial wound healing and defense against pathogens through encapsulation [11,12]. In phagocytosis, bactericidal reactive oxygen species (ROS) are produced, inducing a respiratory burst (RB) that promotes phagocytosis and the elimination of foreign objects [13]. In addition, the intestines are a major organ in the digestive system of shrimps. It is widely known that the activity of digestive enzymes (e.g., trypsin, amylase, lipase, and chymotrypsin) is a crucial indicator of nutrient digestion and uptake in the intestines; this activity is also central to shrimp growth and health [14,15,16].

*Litopenaeus vannamei* (*L*. *vannamei*) is one of the most commercially important species of prawns in aquaculture due to its fast growth, high yield, and high economic value. It accounted for 5.8 million tonnes of global shrimp production in 2020 [17]. However, disease outbreaks caused by intensive aquaculture practices in shrimp farming remain the main obstacle to sustainable food production. To resist diseases, antibiotics are used heavily in the aquaculture industry. This not only results in the formation of drug-resistant strains but also toxic residues in food, ultimately posing a threat to human health due to biomagnification and bioaccumulation [18]. Therefore, improving the immune system and functions of the intestinal digestive enzymes of *L*. *vannamei* through natural food or compounds is essential for sustainable shrimp farming.

This study evaluated the effects of the *Panax notoginseng* water extract (PNWE) on the immune responses and digestive enzyme functions of *L*. *vannamei*. The immune parameters included the total haemocyte count (THC), differential haemocyte count (DHC), which includes granular haemocytes (GC), semi-granular haemocytes (SGC), hialin haemocyte (HC)), phenoloxidase (PO) and RB activity, the phagocytic index (PI), and the phagocytosis rate (PR). In addition, we also measured the activity of the intestinal digestive enzymes (including trypsin, chymotrypsin, amylase, and lipase) and the *Vibrio* numbers and observed the histopathology of the hepatopancreas and intestines.

## 2. Materials and Methods

### 2.1. Litopenaeus vannamei

*L*. *vannamei* with a mean weight of 3.8 ± 0.5 g (mean ± SD) were provided by the Department of Aquaculture, at the National Pingtung University of Science and Technology. They were acclimated in the aquatic animal breeding room of the National Kaohsiung University of Science and Technology for seven days and fed with commercial shrimp feed (Shye-Yih, Kaohsiung, Taiwan) twice a day. According to proximate analysis, the commercial diet (which does not contain *Panax notoginseng* or ginseng related products) had 35% crude proteins, 16% ash, 5% crude fats, and 44% carbohydrates. During the trial, the experimental shrimp (60 shrimp/group) were kept in 250 L tanks containing 200 L of seawater at 15‰ salinity; the water temperature was maintained at 25 ± 1 °C, the pH value at 7.8~8.0, and they were fed twice daily with a commercial shrimp feed (8% of body weight).

### 2.2. Preparation of PNWE

*Panax notoginseng* root powder was purchased from the Zhongxin Pharmaceutical Research Center (Tianjin, China). The root powder was added to sterile water (100 g:1 L, *w*/*v*) and stirred for 24 h at room temperature. The mixture was then centrifuged at 910× *g* for 10 min at 4 °C. The supernatant was collected and filtered through a glass vacuum using a 0.45 μm filter membrane. The filtrate was vacuum evaporated at 40~45 °C and then freeze-dried (LABCONCO, Free20ne^®^ 4.5, Kansas City, MO, USA), thus producing the PNWE at a 51.94% yield. The PNWE samples were stored at −80 °C for future use.

### 2.3. Experimental Design

There were four treatment groups in this study, which were control and different concentrations of the PNWE (50, 100, and 200 μg (g shrimp)^−1^). Approximately 20 μL of the PNWE with a concentration of 9.5, 19, or 38 mg/mL was syringe (25 G × 1″) injected intramuscularly into the second to third abdominal segment of the *L*. *vannamei*, to achieve doses of 50, 100, and 200 μg/g body weight, respectively. The control group consisted of shrimps injected with only saline (20 μL). At each time point (12, 24, 48, 72, 168, and 240 h), ten shrimps were used per treatment from each group, meaning that a total of 250 shrimps were used in this study (four groups × six time points × ten shrimps + ten shrimp (0 h, no treatment served as the initial group)). The haemolymph of the shrimp was sampled to analyze the immune parameters (THC, SGC, GC, HC, PO and RB activity, the PR, and the PI); while the intestines were sampled to measure the activity of the intestinal digestive enzymes (including trypsin, chymotrypsin, amylase, and lipase) as well as the *Vibrio* numbers inside the intestines. The hepatopancreas and intestines were sampled at 240 h for histopathological observations.

### 2.4. Measurement of Immune Parameters

#### 2.4.1. Measurement of THC

The THC measurement method was carried out according to Yeh et al. [19]. The THC was calculated by placing 20 μL of an anticoagulant–haemolymph mixture on a hemacytometer and using an inverted phase-contrast microscope (Olympus IX 71, Tokyo, Japan).

#### 2.4.2. Measurement of DHC

The DHC measurement method was modified by Maftuch et al. [20]. A 100 μL sample of haemolymph was drawn using an injection needle containing 100 μL of 1% glutaraldehyde (Sigma-Aldrich, St. Louis, MO, USA), then mixed with 100 μL of 0.2 M sodium cacodylate buffer. Then, 10 μL of the mixture was taken, spread, and air-dried on a microscope slide, and soaked with 70% methanol (Aencore Chemical, Whitehorse Road, Surrey Hills, Australia) for 10 min for fixation. Afterward, a Giemsa stain (Giemsa: ddH_2_O = 1:20, Riedel-deHaën, Seelze, Germany) was applied to the slide for 10 min. The GC, SGC, and HC percentages were calculated using an upright phase-contrast microscope (H600L, Nikon, Tokyo, Japan).

#### 2.4.3. Measurement of Phagocytic Activity

The phagocytosis measurement method was modified by Jian and Wu [21]. First, 100 μL of haemolymph and 400 μL anticoagulant were mixed. They were then centrifuged at 1258× *g*, 4 °C for 10 min, and the supernatant was removed. Using KC-199 media (Sigma-Aldrich, St. Louis, MO, USA), the haemocyte concentration was determined to be 1 × 10^6^ cells/mL. The haemocytes were then cultivated on microscope slides with cover glasses for an hour at room temperature. Afterward, the unattached haemocytes were then removed using KC-199 media. Then, 1 mL of Latex beads (10^8^ beads/mL with a particle diameter of 1.094 m, Sigma-Aldrich, St. Louis, MO, USA) was added to the slides, which were then cultured for one hour at room temperature. The unattached Latex bead supernatant was washed away using KC-199 medium, and then 200 μL of methanol was added for a five-minute fixation. Afterward, the slides were dyed with 1 mL Giemsa stain (Giemsa:ddH_2_O = 1:20) for 15 min. Haemocyte observations were carried out using an upright phase-contrast microscope (H600L, Nikon, Tokyo, Japan). The phagocytosis-related parameters were calculated using the following equations:

Phagocytic rate (PR) = (no. of cells ingesting beads/no. of cells observed) × 100%

Phagocytic index (PI) = (no. of cells ingesting beads/no. of cells observed) × (no. of beads ingested/no. of cells observed) × 100%

#### 2.4.4. Measurement of RB Activity

The quantity of superoxide anions in haemolymph was used as an indicator of RB. First, 100 μL of poly-L-lysine (0.2%; Sigma-Aldrich, St. Louis, MO, USA) was added to each well of a 96-well plate and left to stand for 90 min at room temperature before the supernatant was removed. For the induced group, 100 μL of 0.1% zymosan (Sigma-Aldrich, St. Louis, MO, USA) was added, while 100 μL of MCHBSS (Sigma-Aldrich, St. Louis, MO, USA) culture was added for the non-induced group. The reactions were conducted for 30 min at 37 °C and were terminated by adding 100 μL methanol. The wells were washed thrice using 100 μL 70% methanol (agitated for 1 min for each wash). A mixture of 120 μL 2 M potassium hydroxide and 140 μL dimethyl sulfoxide (Sigma-Aldrich, St. Louis, MO, USA) was added. The absorbance at 630 nm was measured using an ELISA reader.

RB activity = (induced group − non-induced group)/non-induced group

#### 2.4.5. Measurement of PO Activity

The measurement of the PO activity was as follows: 100 μL of an anticoagulant–haemolymph mixture was added to a 96-well plate and centrifuged at 300× *g* for 20 min at 4 °C, and the supernatant was removed. For the induced group, 100 μL of cacodylate buffer and 100 μL of 0.1% trypsin were added; 200 μL of cacodylate buffer (Sigma-Aldrich, St. Louis, MO, USA) was added for the non-induced group. The reactions were carried out for 10 min. The reaction continued for 5 min at 37 °C with 100 μL of a 0.3% L-3,4-dihydro-xyphenylalanine (Sigma-Aldrich, St. Louis, MO, USA) solution. The absorbance at 490 nm was measured using an ELISA reader.

PO activity = (induced group − non-induced group)/non-induced group

### 2.5. Analysis of Digestive Enzymes

#### 2.5.1. Measurement of Trypsin Activity

The intestines were weighted and homogenized using a tissue homogeneous solution (containing 100 mM Tris-HCl; 0.1 mM ethylenediaminetetraacetic acid; 0.1% Triton X-100, Sigma-Aldrich, St. Louis, MO, USA). The crude enzyme solution was obtained from the supernatant after the solution was centrifuged at 5600× *g* for 30 min at 4 °C. A mixture of 3 mL buffer (containing pH 8.0, 50 mM Tris-HCl, and 20 mM calcium chloride, Sigma-Aldrich, St. Louis, MO, USA) and 0.5 mM N-benzoyl-L-arginine ethylester (BTEE; Sigma-Aldrich, St. Louis, MO, USA) were placed into a cuvette with 100 µL of the crude enzyme solution. The absorbance at 253 nm at the first and third minutes was measured using a spectrophotometer [22]. The equation is as follows:

Trypsin activity (U/mg protein) = [(A3 − A1)/2] *×* 10/P

A1: Absorbance at the first minute; A3: Absorbance at the third minute; P: Protein concentration.

#### 2.5.2. Measurement of Chymotrypsin Activity

Chymotrypsin activity was measured based on the method of Trenzado et al. [22]. A mixture of 1.5 mL buffer and 1.70 mM BTEE was placed into a cuvette with 100 µL of the crude enzyme solution. The absorbance at 256 nm at the first and third minutes was taken using a spectrophotometer. The equation is as follows:

Chymotrypsin activity (U/mg protein) = [(A3 − A1)/2] *×* 10/P

A1: Absorbance at the first minute; A3: Absorbance at the third minute; P: Protein concentration.

#### 2.5.3. Measurement of Amylase Activity

Amylase activity was measured based on the manufacturer’s instructions using a commercial kit (AY1580, Randox Laboratories Ltd., Crumlin, County Antrim, UK). First, 1 mL of R1 solution was placed into a cuvette 20 µL of the crude enzyme solution. The absorbance at 405 nm at the first and third minutes was measured using a spectrophotometer. The equation is as follows:

Amylase activity (U/mg) = (A3 − A1)/2 *×* 4712

A1: Absorbance at the first minute; A3: Absorbance at the third minute.

#### 2.5.4. Measurement of Lipase Activity

Lipase activity measurements were carried out based on the manufacturer’s instructions using a commercial kit (LI188, Randox Laboratories Ltd., Crumlin, County Antrim, UK). First, 1 mL of R1 solution was placed into a cuvette 40 µL of the crude enzyme solution or lipase standard solution. The absorbance at 340 nm at the fourth and ninth minutes was taken using a spectrophotometer. The equation is as follows:

Lipase activity (U/mg) = Sample (A9 − A4) *×* Factor (activity standard/stander (A9 − A4))

A4: Absorbance at the fourth minute; A9: Absorbance at the ninth minute.

### 2.6. Analysis of Vibrio Number

The individual shrimp’s body surface was disinfected with 95% ethanol and then rinsed with sterile distilled water. The intestines were chopped using a pair of scissors until mashed and homogeneous. Then, they were homogenized using 0.85% NaCl (sodium chloride; Sigma-Aldrich, St. Louis, MO, USA) until they were 12.5 times the weight of the intestines. A 100 μL sample was spread on a tryptic soy agar (TSA; Difco, Becton Dickinson, East Rutherford, NJ, USA) containing 1.5% NaCl. After incubation for 24 h at 28 °C, the total number of bacterial colonies was estimated; the effective bacterial colonies were between 25 and 250. The following is the formula:

Bacteria concentration: the number of bacteria colonies × dilution factor × 0.1 mL × the amount of 0.85% NaCl added (mL)

### 2.7. Analysis of Tissue Staining with Hematoxylin-Eosin

The hepatopancreas and intestinal tissues were fixed in 10% neutral-buffered formalin for two days. Then, they were dehydrated progressively with alcohol at different concentration gradients (70%, 95%, 100%) before encapsulation. The paraffin-embedded tissues were cut into 5-µm thick pieces using a cutter. The pieces were then fixed on a microscope slide using protein glycerol and stained through hematoxylin-eosin (H&E) staining. The samples were mounted on the microscope slides using cover glasses, and the prepared tissue samples were observed under an upright phase-contrast microscope (H600L, Nikon, Tokyo, Japan).

### 2.8. Statistical Analysis

Statistical analysis of the experimental data was carried out through analysis of variance (ANOVA) and Duncan’s multiple range tests in SPSS 12.0 software to check for significant differences (*p* < 0.05).

## 3. Results

### 3.1. Effect of the PNWE on the Immune Response of L. vannamei

**Figure 1:**
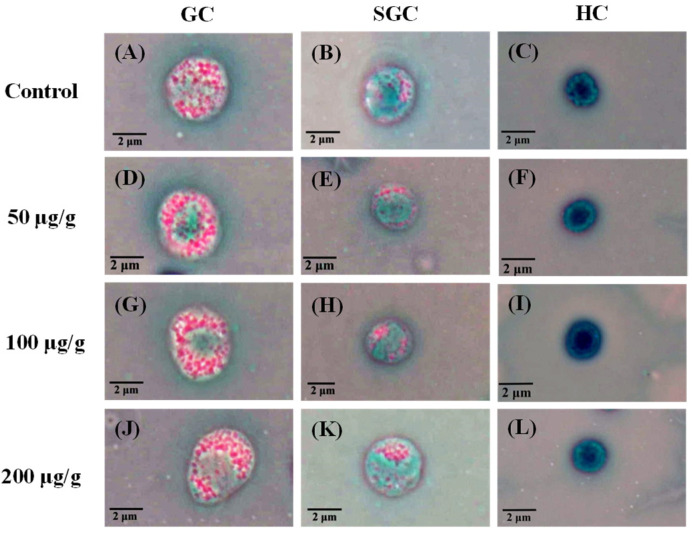
Phase-contrast micrograph of white shrimp that received the PNWE at 50, 100, and 200 µg (g shrimp)^−1^ and control shrimp. (**A**–**C**) control at 240 h; (**D**–**F**) injection 50 µg (g shrimp)^−1^ PNWE at 240 h; (**G**–**I**) injection 100 µg (g shrimp)^−1^ PNWE at 240 h; (**J**–**L**) injection 200 µg (g shrimp)^−1^ PNWE at 240 h. (1000×). PNWE: *Panax notoginseng* water extract. GC: granular haemocyte. SGC: semi granular haemocyte. HC: hialin haemocyte.

**Figure 2:**
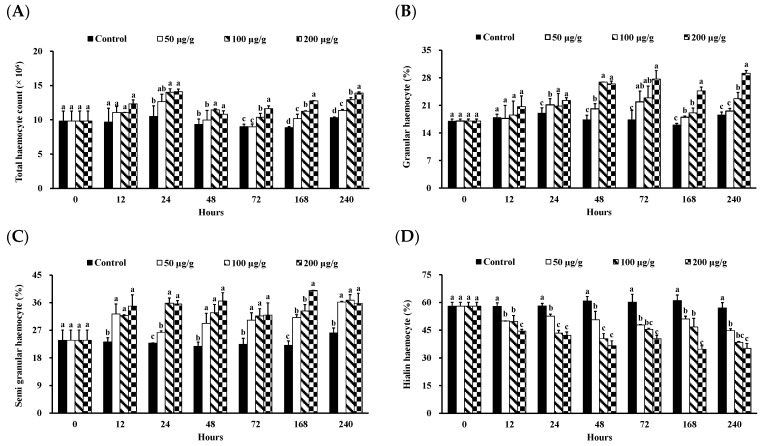
THC (**A**), GC (**B**), SGC (**C**), and HC (**D**) of *Litopenaeus vannamei* that received the PNWE at 50, 100, and 200 µg (g shrimp)^−1^ and control shrimp. Data are expressed as mean ± SD (*n* = 8). Different superscript letters indicate that the same exposure time significantly differs (*p* < 0.05) among groups (Duncan’s multiple range tests). PNWE: *Panax notoginseng* water extract.

There was no mortality of *L. vannamei* in all treatment groups until the 240-h experiment. The morphology and count of haemocytes in the *L*. *vannamei* injected with different concentrations of the PNWE are shown in Figure 1 and Figure 2. Based on upright phase-contrast microscope observations, no deformations or ruptures were observed at 240 h in the GC, SGC, and HC of *L*. *vannamei* injected with different concentrations of the PNWE (Figure 1). From 24 to 72 h, the THCs of *L*. *vannamei* injected with 100 and 200 µg (g shrimp)^−1^ of the PNWE were significantly higher than that of the control (*p* < 0.05; Figure 2A). Finally, from 168 to 240 h, different concentrations of the PNWE significantly increased the THC (*p* < 0.05). From 24 to 240 h, the GC (Figure 2B) and SGC (Figure 2C) percentages of *L*. *vannamei* injected with different concentrations of the PNWE were significantly higher than that of the control (*p* < 0.05), while the HC percentage was significantly lower than the control (*p* < 0.05) (Figure 2D). The most significant increase in the GC percentage was observed in *L*. *vannamei* injected with 100 and 200 µg (g shrimp)^−1^ of the PNWE.

**Figure 3:**
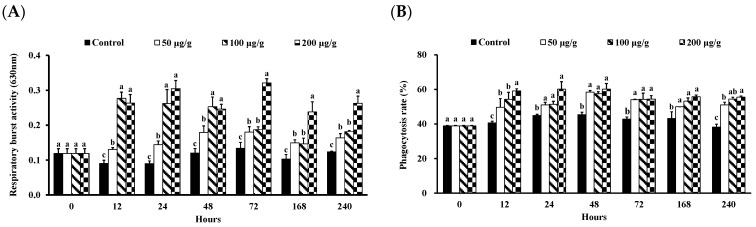

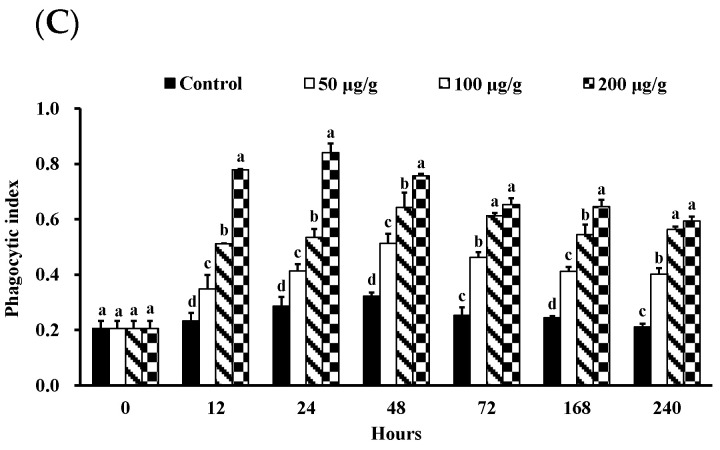
RB (**A**) activity, PR (**B**), and PI (**C**) of *Litopenaeus vannamei* that received the water extract of *Panax notoginseng* at 50, 100, and 200 µg (g shrimp)^−1^ and control shrimp. Data are expressed as mean ± SD (*n* = 8). Different superscript letters indicate that the same exposure time significantly differs (*p* < 0.05) among groups (Duncan’s multiple range tests).

**Figure 4:**
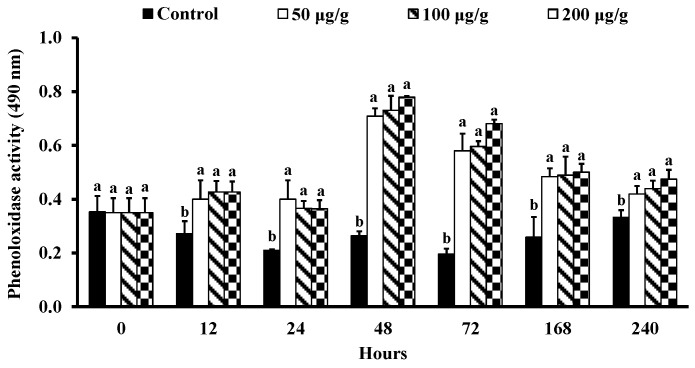
PO activity of *Litopenaeus vannamei* that received the water extract of *Panax notoginseng* at 50, 100, and 200 µg (g shrimp)^−1^ and control shrimp. Data are expressed as mean ± SD (*n* = 8). Different superscript letters indicate that the same exposure time significantly differs (*p* < 0.05) among groups (Duncan’s multiple range tests).

The phagocytosis and PO activity of *L*. *vannamei* injected with different concentrations of the PNWE are shown in Figure 3 and Figure 4. From 12 to 240 h, the RB activity was significantly higher than that of the control in different concentrations of the PNWE (*p* < 0.05) (Figure 3A). The most significant RB activity was observed in *L*. *vannamei* injected with 100 and 200 µg (g shrimp)^−1^ of the PNWE at 12 to 48 h. However, the most significant increase in RB activity was observed in *L*. *vannamei* injected with 200 µg (g shrimp)^−1^ of the PNWE from 72 to 240 h. The PR (Figure 3B) and the PI (Figure 3C) of *L*. *vannamei* injected with different concentrations of the PNWE were significantly higher than that of the control (*p* < 0.05) from 12 to 240 h. The increase in PI was most significant in the group injected with 100 and 200 µg (g shrimp)^−1^ of the PNWE. From 12 to 240 h, the PO activity of *L*. *vannamei* injected with different concentrations of the PNWE was significantly higher than that of the control in different concentrations of the PNWE (*p* < 0.05; Figure 4).

### 3.2. Effect of PNWE on the Digestive Enzyme Functions of L. vannamei

**Figure 5:**
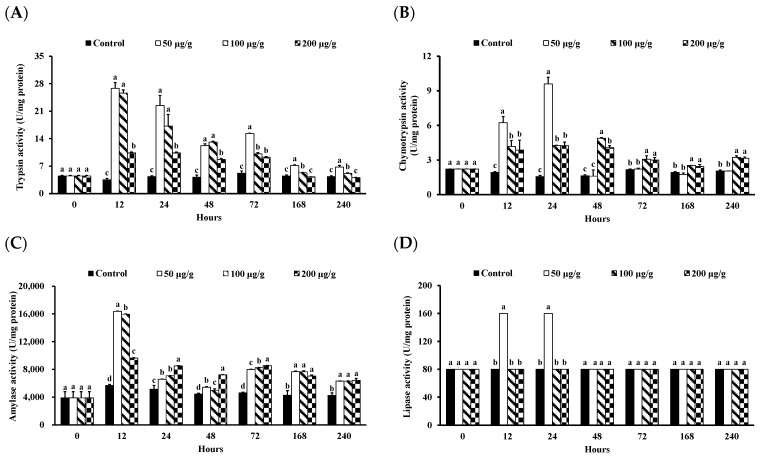
Trypsin (**A**), chymotrypsin (**B**), amylase (**C**), and Lipase (**D**) activity of *Litopenaeus vannamei* that received the water extract of *Panax notoginseng* at 50, 100, and 200 µg (g shrimp)^−1^ and control shrimp. Data are expressed as mean ± SD (*n* = 8). Different superscript letters indicate that the same exposure time significantly differs (*p* < 0.05) among groups (Duncan’s multiple range tests).

The intestinal digestive enzyme activity in *L*. *vannamei* injected with different concentrations of the PNWE is shown in Figure 5. From 12 to 72 h, the trypsin activity of *L*. *vannamei* injected with the PNWE was significantly higher than that of the control (*p* < 0.05). The 50 and 100 µg groups (g shrimp)^−1^ were most significant from 12 to 48 h (Figure 5A). From 148 to 240 h, the trypsin activity of *L*. *vannamei* injected with 50 and 100 µg (g shrimp)^−1^ of the PNWE was significantly higher than that of the control (*p* < 0.05); 50 µg (g shrimp)^−1^ was the most significant. From 12 to 24 h, the chymotrypsin activity of *L*. *vannamei* injected with the PNWE was significantly higher than that of the control (*p* < 0.05; Figure 5B), with the 50 µg (g shrimp)^−1^ group being the most significant. From 48 to 240 h, the chymotrypsin activity of *L*. *vannamei* injected with 100 and 200 µg (g shrimp)^−1^ of the PNWE had the most significant increase in chymotrypsin activity (*p* < 0.05). Similarly, from 12 to 240 h, the amylase activity of *L*. *vannamei* injected with the PNWE was significantly higher than that of the control (*p* < 0.05; Figure 5C), with the 50 µg (g shrimp)^−1^ at 12 h being the most significant. Furthermore, from 24 to 72 h, the 200 µg (g shrimp)^−1^ group had the most significant increase in amylase activity. Finally, from 12 to 24 h, the lipase activity of *L*. *vannamei* injected with 50 µg (g shrimp)^−1^ of the PNWE was significantly higher than that of the control (*p* < 0.05; Figure 5D).

### 3.3. Effect of PNWE on the Variation in Vibrio Number and Histologic Patterns of L. vannamei

**Figure 6:**
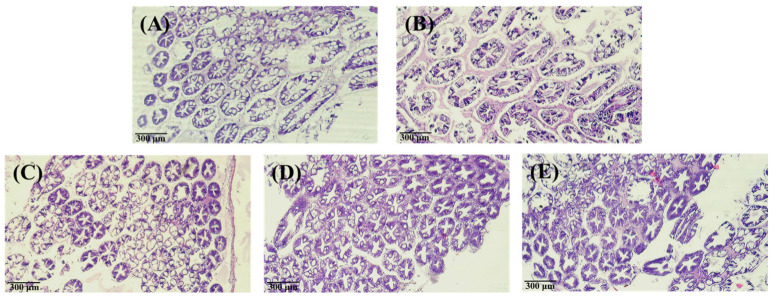
Inverted micrographs of H&E-stained hepatopancreas of white shrimp that containing (**A**) control at 0 h; (**B**) control at 240 h; (**C**) injection 50 µg (g shrimp)^−1^ PNWE at 240 h; (**D**) injection 100 µg (g shrimp)^−1^ PNWE at 240 h; (**E**) injection 200 µg (g shrimp)^−1^ PNWE at 240 h. (100×).

**Figure 7:**
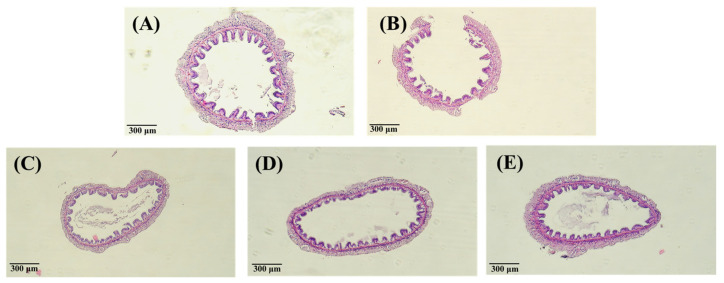
Inverted micrographs of H&E-stained intestine of white shrimp that containing (**A**) control at 0 h; (**B**) control at 240 h; (**C**) injection 50 µg (g shrimp)^−1^ PNWE at 240 h; (**D**) injection 100 µg (g shrimp)^−1^ PNWE at 240 h; (**E**) injection 200 µg (g shrimp)^−1^ PNWE at 240 h. (100×).

As shown in Figure 6 and Figure 7, after 240 h, the hepatopancreas and intestine histology of *L*. *vannamei* injected with different concentrations of the PNWE did not differ significantly from the control.

The variation in the *Vibrio* numbers of *L*. *vannamei* injected with different concentrations of the PNWE is shown in Table 1. At 48 h, the *Vibrio* numbers in the intestines of *L*. *vannamei* injected with 100 and 200 µg (g shrimp)^−1^ of PNWE were significantly lower than that of the control (*p* < 0.05). In addition, from 72 to 240 h, PNWE significantly reduced the *Vibrio* numbers in the intestines (*p* < 0.05).

## 4. Discussion

Some researchers have indicated that THC is an important indicator of a subject animal’s immune strength [23,24]. Several studies have demonstrated that several plants can increase the THC in shrimp; for example, *Astragalus membranaceus* polysaccharide [25], *Eleutherine bulbosa* (Mill.) Urb. [26], *Moringa oleifera* leaf extract [27], *Phyllanthus amarus* extract [28], and *Solanum ferox* and *Zingiber zerumbet* extracts [29]. In this study, the PNWE injection into *L*. *vannamei* increased THC. Research suggests that immunostimulants can increase THC because it prompts a faster maturation of the haemocyte progenitors in haemopoietic tissue, followed by the release of new cells into the circulatory system to maintain the quantity and function of haemocytes in shrimp [28]. Thus, the PNWE increases THC in shrimp, which may be due to the promotion of the maturation of haemocyte progenitor cells in the hemopoietic tissue.

Some plant extracts and Chinese herbal medicines increase the quantity of various types of haemocytes (including SGC, GC, and HC) and strengthen the immune system. Pan et al. (2018) observed that adding *Angelica sinensis* polysaccharide to the feed of *L*. *vannamei* increases SGC, HC, and GC as well as PO activity [30]. Moreover, adding minor bupleurum decoction to the feed of *L*. *vannamei* increases the SGC, HC, and GC as well as RB activity, thereby strengthening the effects of PR and PI [31]. Similarly, adding *Eleutherine bulbosa* (Mill.) Urb. to the feed of *L*. *vannamei* increases the GC/SGC percentage and thereby the PO and RB activity [26]. This study showed that *L*. *vannamei* injected with the PNWE displayed higher GC and SGC levels as well as better PO and RB activity and higher PR and PI levels. Previous studies have indicated the importance of different haemocyte types in immune responses. In particular, GC is chiefly responsible for the activation of the proPO system and participates in phagocytosis, while SGC oversees phagocytosis and participates in proPO system activity [4,18]. Furthermore, PO is a crucial enzyme in the proPO system because it stimulates the production of melanin to prevent pathogen invasions and boosts phagocytosis [4]. The PR and PI are central to phagocytosis, and the RB (which produces bactericidal ROS) that occurs in the process removes foreign pathogens [23,24]. Therefore, we speculate that the PNWE can improve the immunity of shrimp, which may be due to the increase of GC to promote the activation of the proPO system to enhance the effect of PO, and the increase of SGC to further enhance the phagocytosis. Our experimental results showed a positive correlation between the PO activity and phagocytic capacity of PNWE-injected *L*. *vannamei* with the GC and SGC.

The intestines play a crucial role in food storage, digestion, and nutrient absorption. In addition, they also serve as a crucial immunological barrier against toxins and infectious diseases [32]. The administration of Chinese herbal medicine to aquatic animals enhances the activity of intestinal digestive enzymes, thus increasing the digestion and intake of nutrients and promoting health. Previous studies on the intestinal digestive enzymes of *L*. *vannamei* have revealed that complex Chinese herbal medicines improve protease, amylase, and lipase activity as well as chymotrypsin gene expression [6]; *Withania somnifera* aqueous extract improves amylase and lipase activity [33]; *Moringa oleifera* leaf extract improves protease, amylase, and lipase activity [27]; and *Andrographis paniculate* improves trypsin and amylase gene expression [3]. Our results demonstrated that *L*. *vannamei* injected with the PNWE also had better trypsin, chymotrypsin, and amylase activity. Intestinal digestive enzymes are directly associated with nutrient digestion and intake and animal growth performance, for example, trypsin enhances protein hydrolysis, amylase catalyzes the hydrolysis of starch into sugars, and chymotrypsin catalyzes the hydrolysis of protein into smaller peptides [34,35]. Therefore, the PNWE improves the degradation of proteins into smaller peptides and catalyzes the hydrolysis of starch into sugars to maintain the growth performance of shrimp.

Research has shown that different doses of the same material produce different regulating effects. *Lateolabrax japonicas* fed with >15 g/kg of a Chinese herbal medicine mixture can enhance the non-specific immune, while >20 g/kg can promote digestive enzyme expression [36]. After feeding *Oreochromis niloticus* with *Aegle marmelos* fruit extract, >10 g/kg can promote a digestive enzyme expression, while >15 g/kg can enhance the non-specific immune [37]. In addition, *L*. *vannamei* fed with >5 g/kg of complex Chinese herbal medicine can promote a digestive enzyme gene expression, while >10 g/kg can increase THC [6]. This study indicated that, after *L*. *vannamei* were injected with 50, 100, and 200 μg (g shrimp)^−1^ of the PNWE, >50 μg (g shrimp)^−1^ can promote a digestive enzyme expression, while enhancing the immune response would require > 100 μg (g shrimp)^−1^. Therefore, >100 μg (g shrimp)^−1^ of the PNWE simultaneously improves the immune response and digestive enzyme expression of *L*. *vannamei*.

*Panax notoginseng* is a traditional Chinese herbal medicine not only commonly used in human clinical applications but has also been shown to have beneficial effects on fish. *Panax notoginseng* [38] and its active ingredient notoginsenoside R1 [39] can promote angiogenesis in zebrafish. Sun et al. (2018) [9] found that supplementing high lipid diets fed to hybrid groupers with *Panax notoginseng* extract significantly improved their growth performance and feed utilization. Furthermore, the extract regulated immune-related enzyme activity, resulting in an increased immune ability, and improved intestinal morphology. In the present study, shrimp treated with the PNWE showed an increased haemocyte count improving the immune responses. Regulating intestinal digestive enzymes could enhance food assimilation efficiency and shrimp’s nutritional condition. Based on these research results, it can be seen that *Panax notoginseng* enhanced the overall health status in aquatic animals and has the potential to be an alternative preventive treatment.

Studies have confirmed that natural substances have the potential to be used as immunomodulators and growth promoters for aquatic animals, which can be mainly attributed to the active components of natural substances. *Panax notoginseng* saponins are the main active components of the *Panax notoginseng* root. They contain a mixture of dammarane-typ saponins such as *Panax* notoginsenoside R1, ginsenoside Rg1, Rd, and Rb1 [40]. Sun et al. (2018) [9] reported that *Panax notoginseng* extract promoted the growth of hybrid groupers (*Epinephelus lanceolatus* ♂ × *Epinephelus fuscoguttatus* ♀) as well as their antioxidant capacity, mainly because the active components were influenced by the *Panax notoginseng* saponins [9]. Our results indicated that the PNWE improved the immune response and digestive enzyme activity of *L*. *vannamei*, which may be attributed to the *Panax notoginseng* saponins of *Panax notoginseng*. We previously demonstrated that the PNWE contains 13.98 ± 2.60 mg/g of notoginsenoside R1 and 34.67 ± 5.51 mg/g of ginsenoside Rg1 [41].

## 5. Conclusions

The PNWE enhances the immune responses of *Litopenaeus vannamei* by increasing HC, PO, RB, and phagocytic activity while sustaining their growth performance by regulating the activity of intestinal digestive enzymes (trypsin, chymotrypsin, amylase, and lipase). Therefore, the PNWE is a promising immunomodulator for shrimp with the potential to facilitate the sustainable development of the aquaculture industry in the future.

## Figures and Tables

**Table 1:** *Vibrio* numbers of *Litopenaeus vannamei* that received the water extract of *Panax notoginseng* at 50, 100, and 200 μg (g shrimp)^−1^ and control shrimp.

Group (µg (g shrimp)^−1^)	*Vibrio* Numbers (×10^7^) after Time Elapsed						
	0	12	24	48	72	168	240
0	12.23 ± 2.56 ^a^	10.14 ± 0.43 ^a^	11.26 ± 0.16 ^a^	10.13 ± 0.36 ^a^	10.48 ± 5.59 ^a^	10.25 ± 3.25 ^b^	10.16 ± 0.21 ^a^
50	12.23 ± 2.56 ^a^	8.30 ± 1.38 ^a^	7.03 ± 0.96 ^a^	7.68 ± 1.27 ^ab^	3.66 ± 1.17 ^b^	2.64 ± 1.15 ^b^	4.73 ± 1.68 ^b^
100	12.23 ± 2.56 ^a^	8.07 ± 0.30 ^a^	6.78 ± 1.52 ^a^	4.17 ± 0.29 ^c^	3.69 ± 0.36 ^b^	1.70 ± 0.06 ^b^	2.92 ± 0.30 ^b^
200	12.23 ± 2.56 ^a^	10.55 ± 3.97 ^a^	8.05 ± 2.57 ^a^	6.56 ± 1.51 ^bc^	2.27 ± 0.74 ^b^	1.20 ± 0.10 ^b^	2.56 ± 0.56 ^b^

Data are expressed as mean ± SD (*n* = 8). Different superscript letters indicate significant differences (*p* < 0.05) between the same column (Duncan’s multiple range tests).

## Data Availability

Data are available from the corresponding author upon reasonable request.
